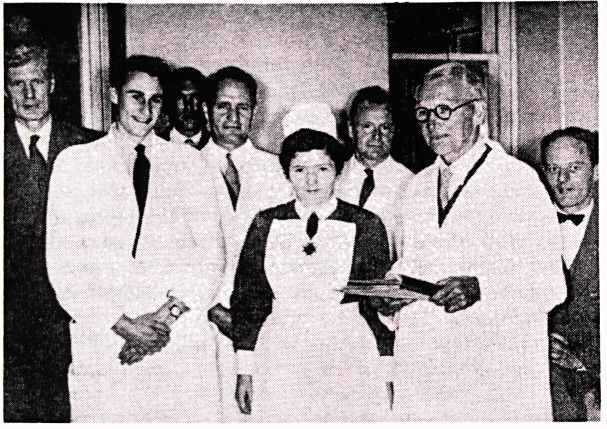# Arthur Wilfrid Adams

**Published:** 1975

**Authors:** 


### Arthur Wilfrid Adams

Arthur Wilfrid Adams died on 9th December, 1974, aged 82 years. He was a Bristolian, the son of Avery Adams, who was for many years Secretary to Bristol Education Committee and a well known local artist. Wilfrid was educated at Clifton College and then studied medicine in Bristol and at the London Hospital qualifying M.B., B.S. (London) in 1916. After service with the RAMC in the First World War he proceeded to F.R.C.S.(Eng.) in 1919 and M.S.(Lond.) in 1921.

Wilfrid's brilliance was quickly recognised so that he became Honorary Assistant Surgeon to the Bristol Royal Infirmary in 1922 and soon after to the Bristol Royal Hospital for Sick Children and Women. When Bristol Health Committee acquired Southmead Hospital for development as an acute general hospital he was appointed Consultant Surgeon in 1933 and played a leading rôle in the progress of the surgical service and in particular laid the foundations for the Regional Urological Department which was subsequently based at that hospital.

He particularly enjoyed surgery for children and was enthusiastic to develop an efficient modern Children's Hospital in Bristol. His technique and versatility were such that in the 1930s he performed with success several lobectomies for bronchiectasis, the removal of a pituitary adenoma and frequently deputised for the many paediatric E.N.T. emergencies before the era of antibiotics when the difficult diagnosis and surgical treatment of the intracranial complications of sepsis were commonplace. Treatment of congenital abnormalities especially clubfoot, cleft lip and palate, and infantile hypertrophic pyloric stenosis presented a special challenge and there are successful citizens today whose gratitude is unceasing for the care he gave them in infancy.

At the height of his career, immaculately dressed with bowler hat and white chamois gloves he travelled in a chaffeur-driven car all over the West Country for consultations and much farther afield to other teaching centres to keep abreast of the latest surgical developments; a box file always accompanied him which contained detailed and beautifully illustrated notes of his patients.

As a perfectionist, who strove for ever higher standards, he was often the first to introduce new techniques into the surgical practice of the Infirmary. The scarcity of skilled anaesthetists stimulated him to administer spinal anaesthesia himself for much of his abdominal and urological surgery. His delightful use of eponyms enlivened many a difficult operating session and his enthusiasm stimulated his house officers "To go the second mile" and to work indefatigably in such trying tasks as intravenous drip therapy with makeshift equipment and frequent use of blood transfusion which involved finding and bleeding the donors, as well as cross-matching the blood. Though he was an exacting Chief, young doctors and budding surgeons regarded him with great affection and gratitude for the encouragement and training he gave them at the start of their careers.

Before many of his colleagues, Wilfrid Adams saw the need for the specialist surgeon so his interests gradually focused into Urology. The Department he created in Bristol is a permanent memorial to his devotion and tenacity and is second to none in the country today. As a pioneer in this field he was a founder member of the British Association of Urological Surgeons and was honoured with the Presidencies of the Moynihan Chirurgical Club and the Urological Section of the Royal Society of Medicine. He also wrote some notable articles on Urology and other surgical topics. He was always a loyal supporter of the Bristol Medico-Chirurgical Society and in his earlier years contributed frequently to the meetings which he continued to attend regularly until shortly before his death.

Obsessional about his own fitness for his task he was a teetotaller, non-smoker and later developed many dietetic eccentricities. Cycling from Bristol to Weston-super-Mare for his operating sessions during World War II, tennis parties on the fine hard court he built in Rodney House garden, and later running round the Downs without socks before breakfast in bitter winter weather were part of his routine programme.

The spacious grounds of Southmead Hospital with easy access to open air for staff and patients, and freedom from the noise and smoke of the centre of the city immediately appealed to him with his love of the countryside and convinced him that this was the right environment for seriously ill patients. It was therefore inevitable that his well-known campaign should begin in the mid-1930s for the new Bristol teaching hospital and medical school to be built in the beautiful grounds of Ashton Court. This became his theme song which at the time fell largely on deaf ears, but young colleagues today are more inclined to regard his "castle in the air" as an exceptional opportunity lost.

The study of nature gave him lifelong enjoyment which he pursued with characteristic energy and eye for detail in his retirement. The seashore, many varieties of wild birds, the night sky, trees, plants and flowers always enthralled him with the eye of the artist for beauty of shapes, patterns and colour. He was a philosopher pondering on the wonders of creation and a relentless seeker for the God whom he believed with certainty lay beyond natural phenomena.

Criticism, and even at times ridicule, was inevitable of one who was so far ahead of his time and so tenacious in the pursuit of his ideas and ideals. Invariably he met this with equanimity and as a perfect gentleman he continued to smile without reproach or anger, but was never deterred and always had the courage of his convictions.

Wilfrid married Hilda Ewins who was the first Bristol woman medical graduate and the first woman house officer at Bristol Royal Infirmary. Unfortunately otosclerosis made her severely deaf after the birth of their third child. She died in 1972.

Tragedy came when their only son contracted severe poliomyelitis as he entered his first term at Cambridge University and after a long courageous struggle, in which Hilda devoted her whole life to his care, died relatively young. Wilfrid Adams is succeeded by his two daughters, one of whom is a medical graduate and works in Bristol University Medical Library. The other is married to an Agricultural Economist of the United Nations Organisation. His six fine grandchildren gave him much pleasure and interest in his later years.

## Figures and Tables

**Figure f1:**